# The Clinical Correlation of Regulatory T Cells and Cyclic Adenosine Monophosphate in Enterovirus 71 Infection

**DOI:** 10.1371/journal.pone.0102025

**Published:** 2014-07-10

**Authors:** Shih-Min Wang, I-Chun Chen, Yu-Ting Liao, Ching-Chuan Liu

**Affiliations:** 1 Department of Emergency Medicine College of Medicine, National Cheng Kung University and Hospital, Tainan, Taiwan; 2 Department of Pediatrics, College of Medicine, National Cheng Kung University and Hospital, Tainan, Taiwan; 3 Center of Infectious Disease and Signaling Research, National Cheng Kung University, Tainan, Taiwan; 4 Institute of Basic Medical Sciences, College of Medicine, National Cheng Kung University, Tainan, Taiwan; University of Illinois at Chicago, United States of America

## Abstract

**Background:**

Brainstem encephalitis (BE) and pulmonary edema (PE) are notable complications of enterovirus 71 (EV71) infection.

**Objective:**

This study investigated the immunoregulatory characterizations of EV71 neurological complications by disease severity and milrinone treatment.

**Study Design:**

Patients <18 years with virologically confirmed EV71 infections were enrolled and divided into 2 groups: the hand, foot, and mouth disease (HFMD) or BE group, and the autonomic nervous system (ANS) dysregulation or PE group. Cytokine and cyclic adenosine monophosphate (cAMP) levels, and the regulatory T cell (Tregs) profiles of the patients were determined.

**Results:**

Patients with ANS dysregulation or PE exhibited significantly low frequency of CD4^+^CD25^+^Foxp3^+^ and CD4^+^Foxp3^+^ T cells compared with patients with HFMD or BE. The expression frequency of CD4^−^CD8^−^ was also significantly decreased in patients with ANS dysregulation or PE. Among patients with ANS dysregulation or PE, the expression frequency of CD4^+^Foxp3^+^ increased markedly after milrinone treatment, and was associated with reduction of plasma levels IL-6, IL-8 and IL-10. Plasma concentrations of cAMP were significantly decreased in patients with ANS dysregulation or PE compared with patients with HFMD or BE; however, cAMP levels increased after milrinone treatment.

**Conclusions:**

These findings suggested decreased different regulatory T populations and cAMP expression correlate with increased EV71 disease severity. Improved outcome after milrinone treatment may associate with increased regulatory T populations, cAMP expression and modulation of cytokines levels.

## Introduction

Human enterovirus 71 (EV71), a member of the species human enterovirus A, has emerged as a major nonpolio enterovirus with the potential to cause severe neurological disease in large-scale outbreaks in the Asia Pacific area [1]. In children, EV71 infection manifests most frequently as hand, foot, and mouth disease (HFMD) or herpangina, both of which are self-limiting conditions. Young children under 3 years of age are particularly susceptible to the most severe forms of EV71-associated neurological disease, including brainstem encephalitis (BE), and poliomyelitis-like paralysis [2], [3]. EV71-related BE is classified into 3 critical stages according to disease severity: uncomplicated BE, autonomic nervous system (ANS) dysregulation, and pulmonary edema (PE) [1], [4].

Without milrinone treatment, patients with PE have a fatality rate as high as 80%–90%. Most patient fatalities occurred within 6–12 hours without prompt care [5]. Pure inotropic agents are associated with worse outcomes, whereas treatment with milrinone, which has both inotropic and vasodilatory effects, is associated with lower mortality [5]. Milrinone increases cardiac output, and reduces systemic vascular resistance and pulmonary capillary wedge pressure without excessively increasing myocardial oxygen consumption [6]. It is a cyclic nucleotide phosphodiesterase (PDE) inhibitor subtype III, and elevates intracellular cyclic adenosine monophosphate (cAMP). PDE3 inhibitors can attenuate inflammation [7], reduce edema formation [8], improve endothelial function [9] and induce pulmonary vasodilation [10].

The role of cAMP as a second messenger in cells in the immune system has been widely described. cAMP exerted inhibitory effects on a variety of components involved in cell activation [11]. The modulating effect of PDE inhibitors on lymphocyte subpopulations and the humoral immune response is likely related to their influence on the synthesis and release of cytokines [12]. PDEs, which are enzymes that accelerate the turnover of cAMP, are upregulated in activated helper T cells [13] and downregulated in regulatory T cell (Tregs) cells [14]. Vendetti et al. showed that CD4 T lymphocytes exert regulatory functions through the release of extracellular cAMP, and that the cyclic nucleotide acts as a primary messenger, which could play a role in the biological modulation of immune responses [15].

Studies of several viruses have demonstrated that Tregs might affect the magnitude of immunity and the outcome of infection. Two broad categories of Tregs have been described. The first is the thymus-derived, naturally occurring Tregs in the CD4^+^CD25^+^ subset present in healthy adult humans [16]. In addition, inducible Tregs generated following a variety of antigenic stimulatory regimes have been described [17]. The forkhead (winged-helix) transcription factor forkhead box P3 (FOXP3) has been suggested to represent a reliable intracellular marker for naturally occurring Tregs [18]. CD4^+^Foxp3^+^ Treg cells are a dedicated population of cells that maintain self-tolerance and immune homeostasis. Certain cytokines amplify the number or activity of Tregs, and most cytokines exert a positive stimulating or supportive effect, although some downregulate Tregs [19].

The modulation of immune cell activation is essential for immunoregulation and resolving inflammation. This study aimed to explore the various Treg populations and cAMP expression in EV71-infected patients by disease severity; and to elucidate the changes of Treg populations, cAMP expression and plasma level of cytokines in response to milrinone treatment in severe EV71 infection.

## Methods

### Case enrollment

The enrolled patients were admitted to National Cheng Kung University Hospital (Tainan, Taiwan) during major EV71 epidemics in 2008. Patients were divided into 2 groups by disease severity: (1) the HFMD or BE group, and (2) the ANS dysregulation or PE group. The records of the identified EV71-infected patients were reviewed for demographic data, clinical features, laboratory findings, neuroimaging manifestations, and outcomes.

The study protocol was approved by the Clinical Research Ethics Committee of National Cheng Kung University Hospital. Written informed consent was obtained from the participants’ parents or guardians.

#### Peripheral blood mononuclear cell preparation and flow cytometry analysis

**Figure 1:**
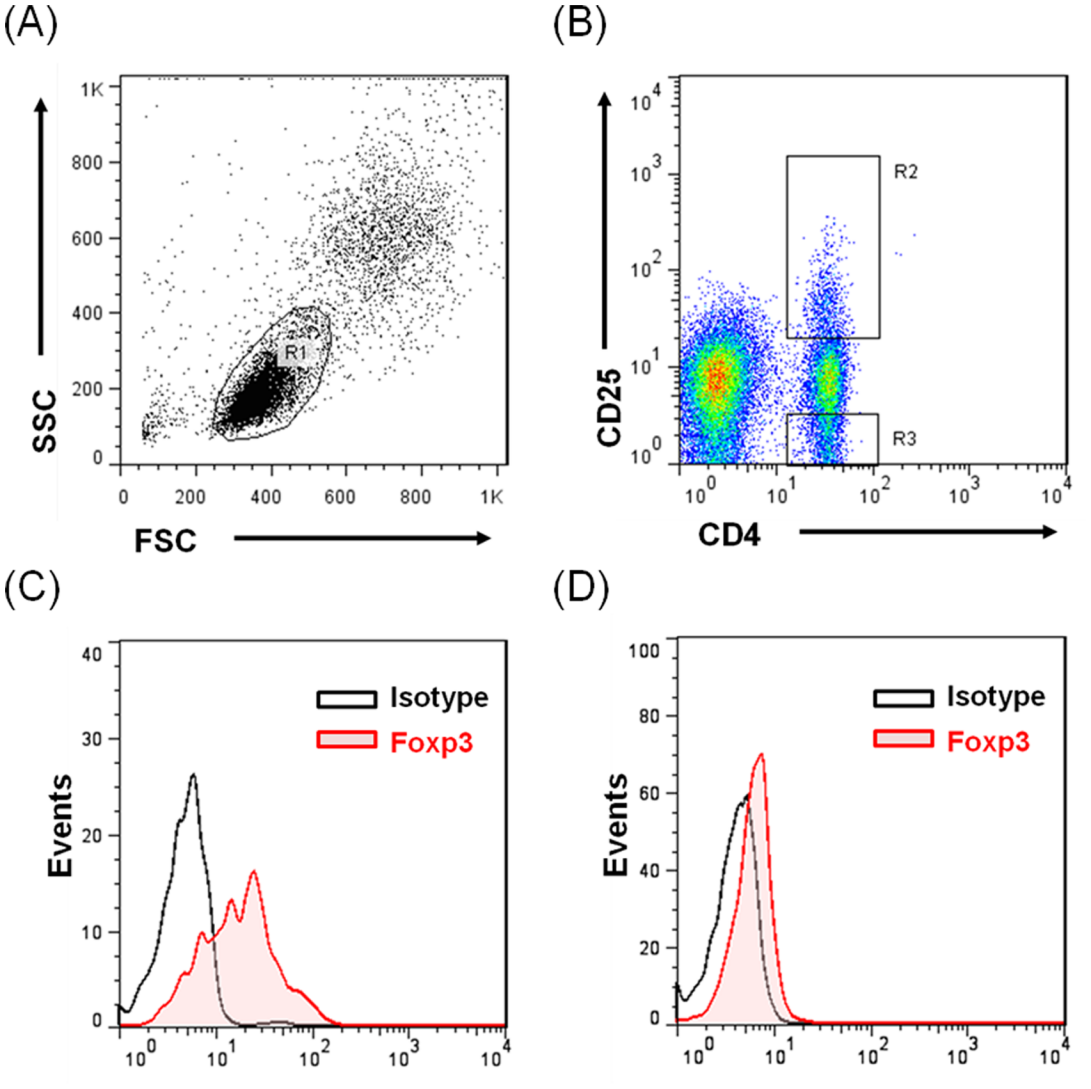
Representative plot illustrating gating strategy. (A) Lymphocytes were gated according to forward and side scatter (R1), (B) CD4^+^CD25^+^ lymphocytes (R2), CD4^+^CD25^−^ lymphocytes (R3). From the gated lymphocyte population, the total Foxp3 expression and isotype control were analyzed (C) (R2) and (D) (R3).

Peripheral blood mononuclear cells (PBMCs) were extracted from peripheral blood by using standard Ficoll density-gradient centrifugation, and were immediately tested using fluorescence activated cell sorter (FACS) analysis. The cells were then washed with PBS and fixed with 0.5 mL of 0.1% glutaraldehyde solution in PBS. Stained lymphocytes were analyzed using flow cytometry (Becton Dickinson Immunocytometry Systems). Data were acquired and analyzed using Cell Quest software (Becton Dickinson). The following fluorescent monoclonal antibodies (MAbs) were used in staining and sorting: peridinin chlorophyll protein-conjugated Leu 4 (CD3 T cells and pan T), phycoerythrin-conjugated Leu-3a (CD4 T cells), Leu-2a (CD8 T cells and NK cells), Leu-11c (CD16 T cells and NK lymphocytes), Leu-19 (CD56 T cells, NK lymphocytes, and a T lymphocyte subset), CD25 (M-A251), and CD127 according to the manufacturer’s instructions (R&D Systems, Minneapolis, MN, USA). FACS intracytoplasmic staining with anti-Foxp3 antibodies demonstrated that CD4^+^ Tregs exhibited Foxp3 expression (eBioscience, San Diego, CA, USA). The gating strategy for each population is shown in Fig. 1.

### Measurement of cytokines

Plasma for cytokine determination was harvested from the participants within 30 minutes of venipuncture from EDTA-anticoagulated venous blood samples, and was stored at −70°C until being analyzed. The cytometric bead array assay (CBA) (BD Pharmingen, CA, USA) consisted of 6bead populations with distinct fluorescence intensities. Concentrations of IL-1β, IL-6, IL-8, IL-10, IL-12 and tumor necrosis factor (TNF)-α were measured using human Th1/Th2 cytokine CBA kits. An ELISA was used for the quantitative determination of TGF-β (Quantikine; R&D Systems). The detection limits for the immunoassays were: 1 pg/mL for IL-1β, 10 pg/mL for IL-6, 10 pg/mL for IL-8, 2.8 pg/mL for IL-10, 2.8 pg/mL for IL-12, and 2.8 pg/mL for TNF-α.

### Measurement of cyclic adenosine monophosphate

To assess cytosolic cAMP concentrations, plasma and CSF were collected from the patients within 30 minutes and were stored at −70°C until analyzed. A cAMP EIA kit (IBL Immuno-Biological Laboratories, Hamburg, Germany) was performed according to the manufacturer’s instructions, and a detection limit of 4 pmol/mL was used.

### Virological Studies

All specimens (throat and rectal swabs, stool, and cerebrospinal fluid) were collected in viral transport media and inoculated onto monolayers of RD, A549, green monkey kidney cells, and Vero cells within 24 hours of collection. The cultures were incubated stationary at 37°C and inspected daily for the viral cytopathic effect (CPE) for a minimum of 14 days. When typical enteroviral CPE was observed, EV71 identification was performed using 2 EV71 type-specific mAbs, 3323 and 3324 (Chemicon, International Inc., Temecula, CA, USA), by using immunofluorescence stains. Because EV71 mAb 3323 cross-reacts with coxsackievirus A16, and mAb 3324 does not cross-react with coxsackievirus A16, EV71 was identified when both mAbs revealed positive staining. These isolates were further confirmed using a neutralization test by using polyclonal antibodies produced in rabbits against EV71.

### Statistical analysis

Proportional data were tested using χ^2^ or Fisher’s exact test. Continuous data were tested using Student’s *t* test. The Mann-Whitney *U* test was used to analyze nonparametric data that did not have a normal distribution. The Wilcoxon signed -rank test was used to analyze non-parametric and paired data. All analyses were performed using SPSS software (version 18.0; Chicago, IL, USA). Data are presented as the mean ± standard deviation (SD), and *P<*.05 was considered statistically significant.

## Results

### Demographic data and laboratory findings

Forty-one patients with either HFMD or BE, or ANS dysregulation or PE, were enrolled in the study. The demographic and laboratory data are summarized in Table 1. The patients were divided into 2 groups: 24 patients were assigned to the HFMD or uncomplicated BE group, and 17 patients were assigned to the ANS dysregulation or PE group. With the exception of CSF lactate levels, which were significantly higher in patients with ANS dysregulation or PE than in patients with uncomplicated BE or HFMD (*P* = .012), all other laboratory characteristics were similar between the 2 groups.

**Table 1:** The basic laboratory findings of enterovirus 71 infection by disease severity.

Characteristics	HFMD+Uncomplicated BE	ANS dysregulation+PE	P
	Mean ± SD	Mean ± SD	
Age (yr)	3.3±2.7	2.4±1.8	0.347
WBC, cells/mm^3^	11736±4539	12769±4172	0.189
Plt, cells/mm^3^	346.2±106.1	364.3±108.1	0.552
CRP, mg/L	11.6±6.5	23.3±37.0	0.318
Glu, mg/dL	112.2±50.0	143.3±123.5	0.554
CK, U/L	79.6±32.1	110.1±101.8	0.968
CK-MB, U/L	3.6±1.8	8.4±9.7	0.43
TnT, ng/mL	0.01±0.001	0.31±0.49	0.41
CSF analysis			
WBC, cells/mm^3^	70.3±105.8	183.7±202.4	0.11
TP, mg/dL	42.7±23.3	55.0±24.8	0.108
Lactate, mmol/L	1.3±0.4	2.8±3.9	0.012
Glucose, mg/dL	63.4±21.8	66.0±22.3	0.242

ANS, autonomic nervous system; CK, creatine kinase; CRP, C-reactive protein; CSF, cerebrospinal fluid; HFMD; hand, foot, and mouth disease; Plt, platelet; PE, pulmonary edema; TnT, troponin T; TP, total protein; WBC, white blood cell.

### Immunophynotype expression analyzed by disease severity

**Figure 2:**
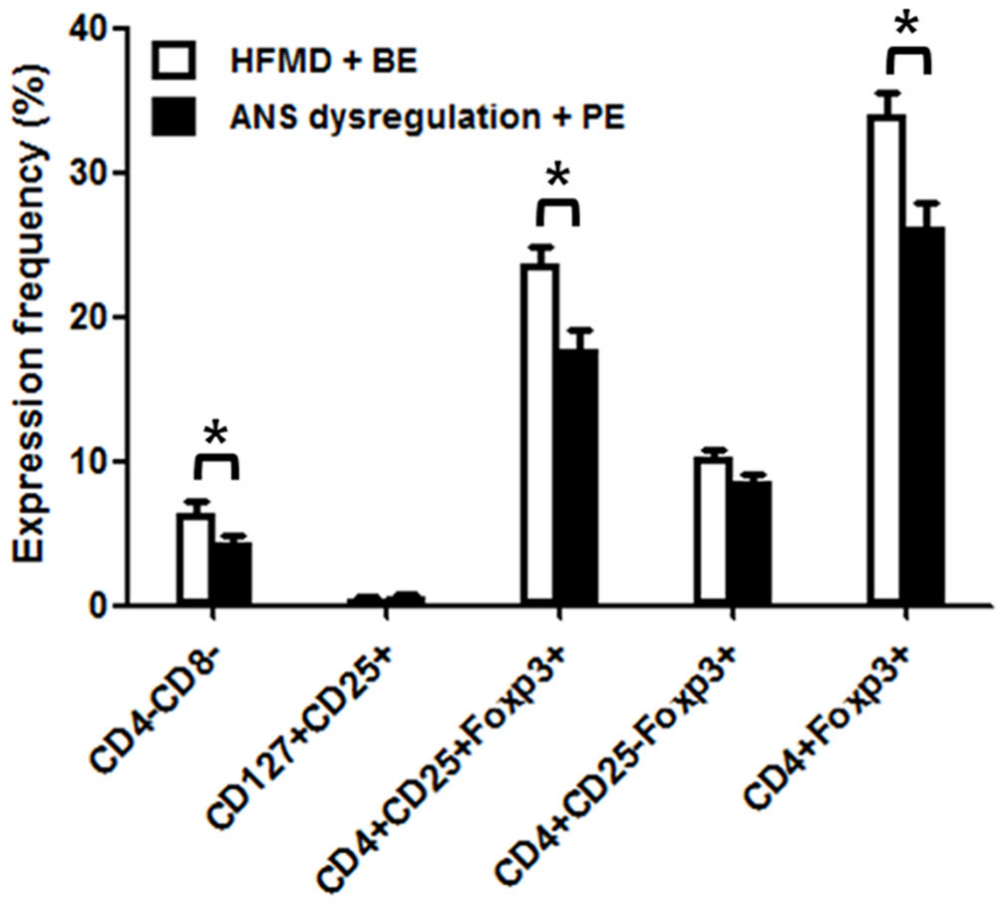
The expression frequency of immunophenotypes in patients with enterovirus 71 infection by disease severity. The expression frequency of CD4^−^CD8^−^, CD4^+^Foxp3^+^ and CD4^+^CD25^+^Foxp3^+^ decreased markedly in patients with autonomic nervous system dysregulation or pulmonary edema, *P<.05.

The expression frequency of CD4^−^CD8^−^ (*P* = .045) decreased significantly in patients with ANS dysregulation or PE (6.4±2.3% vs 4.3±0.4%). No significant differences were observed between the 2 groups in the expression frequency of CD127^+^CD25^+^ (0.4±0.2% vs 0.6±0.1%, *P* = .282) or CD4^+^CD25^−^Foxp3^+^ (10.2±0.6% vs 8.6±0.5%, *P* = .123). However, the expression frequency of CD4^+^Foxp3^+^ (33.9±1.7% vs 26.3±1.7%, *P* = .007) and CD4^+^CD25^+^Foxp3^+^ (23.6±1.2% vs 17.7±1.4%, *P* = .007) decreased significantly in the advanced stages of the disease (ie, in patients with ANS dysregulation or PE) (Figure 2).

### Changes in CD4^+^Foxp3^+^expression and cytokine profiles after milrinone treatment

**Figure 3:**
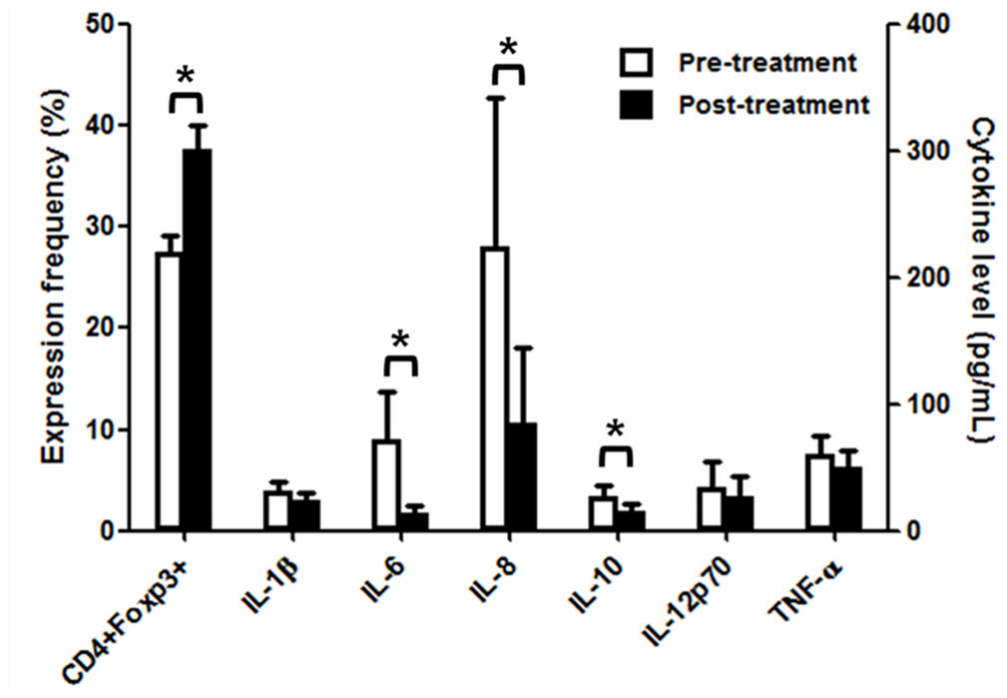
The expression of CD4^+^Foxp3^+^ and cytokine profile in patient with ANS dysregulation or pulmonary edema by milrinone treatment. The expression frequency of CD4^+^Foxp3^+^ increased and plasma cytokine levels of IL-6, IL-8 and IL-10 decreased after milrinone treatment. *P<.01.

Changes of CD4^+^Foxp3^+^ expression and cytokine profiles after milrinone treatment in patients with ANS dysregulation or PE (n = 16) are illustrated in Figure 3. The expression frequency of CD4^+^Foxp3^+^ increased significantly after milrinone treatment (27.6±4.7% vs 37.7±6.5%, *P = *.006) in patients with ANS dysregulation or PE. Plasma levels of IL-6 (72.1±37.7 pg/mL vs 14.7±5.7 pg/mL, *P* = .001), IL-8 (224.7±117.8 pg/mL vs 84.7±59.9 pg/mL, *P* = .002) and IL-10 (27.2±9.0 pg/mL vs 15.6±6.2 pg/mL, *P* = .004) decreased significantly after milrinone treatment. Levels of other cytokines, such as IL-1β (30.8±7.5 pg/mL vs 24.2±6.1 pg/mL, *P* = .14), IL-12 (34.7±19.4 pg/mL vs 26.7±16.7 pg/mL, *P* = .064), and TNF-α (60.1±15.0 pg/mL vs 50.0±13.6 pg/mL, *P* = .109), did not significantly change after milrinone treatment.

### Cyclic adenosine monophosphate expression

**Figure 4:**
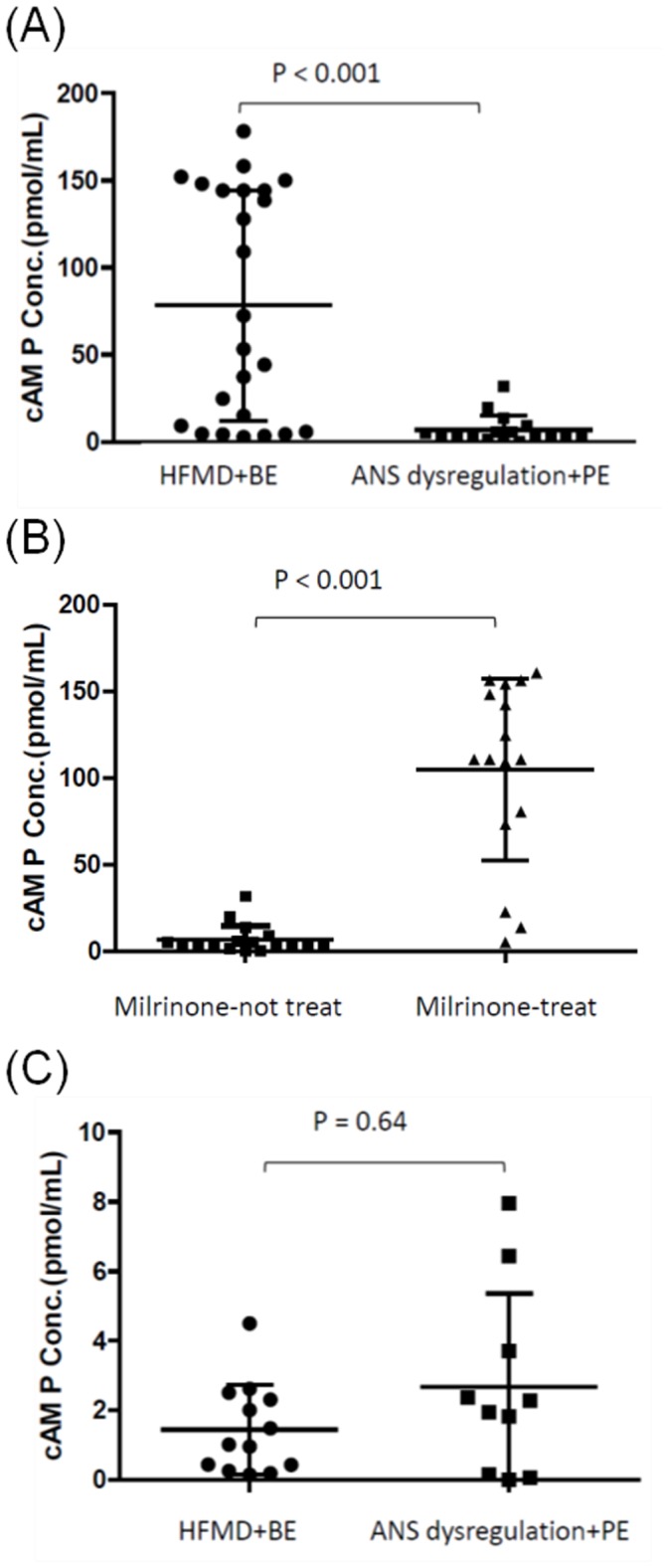
The cyclic AMP levels of enterovirus 71-infected patients in plasma by (A) disease severity and (B) milrinone treatment, and (C) in CSF by disease severity.

Plasma levels of cAMP were significantly lower in patients with ANS dysregulation or PE compared with patients with HFMD or uncomplicated BE (6.9±8.1 pg/mL vs 78.2±65.9 pmole/mL, *P*<.001) (Fig. 4A). In patients with ANS dysregulation or PE who received milrinone treatment, the plasma levels of cAMP significantly increased after treatment (6.9±8.1 pg/mL vs 104.9±52.4 pmole/mL, *P*<.001) (Fig. 4B). Mean CSF cAMP levels did not differ between the HFMD or uncomplicated BE and the ANS dysregulation or PE groups (1.46±1.29 pg/mL vs 2.68±2.69 pg/mL, *P* = .64) (Fig. 4C).

## Discussion

Immunopathogenesis has been shown to play a major role in preventing or contributing to the deleterious effects of excessive immune stimulation that result in fatality in severe EV71 infection [1], [4], [5]. Tregs might influence whether a patient develops severe clinical EV71, manifested in ANS dysregulation or PE. Tregs control the expansion of effector T cells that respond to self and nonself antigens through a variety of mechanisms. T cells with regulatory functions carry either CD4^+^ or CD8^+^ coreceptors, likely act synergistically, as suggested by a vast number of animal and human model studies [20], [21]. The CD4^+^CD25^+^population, which appear to play an active role in downregulating the pathogenic response, is the most characterized example [22]. Foxp3 is a crucial regulator, however, and it has been shown that expression of Foxp3 alone is insufficient to confer regulatory activity. Furthermore, the significantly upregulated CD127 expression of Tregs during in vitro and in vivo activation was demonstrated by an enhanced survival capacity [23]. Other Treg cells, including CD4^−^CD8^−^ double-negative (DN) T cells, were also demonstrated to play an essential role in down regulating the immune response [24]. Therefore, we selected the above Treg profile to study EV71 infection in this study.

In this study, patients with severe EV71 infections exhibited decreased levels of CD4^+^CD25^+^Foxp3^+^, CD4^+^Foxp3^+^, and CD4^−^CD8^−^ T cells compared with patients with mild EV71 infections. Fu and colleagues also reported the proportion of CD4^+^CD25^+^Foxp3^+^ cell and mRNA level of Foxp3 were decreased with deterioration of EV71 disease [25]. However, in addition to CD4^+^CD25^+^Foxp3^+^, CD4^+^Foxp3^+^, CD4^−^CD8^−^ DN T cells and CD127 T cells were explored in current study. Oldenhove et al. [26] reported that Treg cell numbers collapsed by multiple pathways, including the blockade of Treg cell induction and the disruption of endogenous Treg cell homeostasis in a fatal infection model. In previous studies, the elevation of plasma levels of IL-1β, IL-6, IL-8, IL-10, TNF-α and IFN-γ in patients with ANS dysregulation or PE were observed [4], [5], [27], [28]. Fu et al. suggested the decreased CD4^+^CD25^+^Foxp3^+^ expression frequency in severe EV71 infection may associate with the decreased plasma level of TGF-β and increased plasma level of IL-6 [25]. Furthermore, Treg cells express high levels of the Th1 transcription factor T-bet. T-bet expression resulted in IFN-γ production. Treg cells that can also suppress antigen-specific responses in the blood of acutely dengue infected patients. Treg have an impact on the expression of IFN-γ, TNF-α, and IL-6 production in response to dengue antigens in vitro [29]. A mechanism for Th1 cell pathogenicity extending beyond the proinflammatory program to limit Treg cell survival was revealed, and also partially explained above findings.

DN Treg cells are a novel subset of Treg cells. T cells were first identified by Strober et al., [30] who demonstrated that DN T cells have an immunoregulatory function by observing their mediation of suppressor activity in a mixed-lymphocyte reaction. In humans, DN T cell numbers increase during staphylococcal toxic shock syndrome [31] and HIV infection [32] and respond to lipid *Mycobacterium tuberculosis* antigens [33]. DN T cells contribute to the production of IL-17A and IFN-γ in the lungs during inhalational *Francisella* infection, and these cytokines additively activate host cells to control the intercellular growth of live vaccine strains [34]. However, we found DN T cells decreased in severe EV71-infected group. These findings may associate with different cytokines production in various infections.

The effectiveness and efficacy of milrinone treatment in patients with EV71-related PE has been proven in historically controlled [5] and randomized controlled studies [35]. The influence of selective PDE4 inhibitors and nonselective PDE inhibitors on the activity of immune cells, including lymphocytes, has been studied extensively [36], [37]. This study demonstrated the intervention of milrinone therapy not only increased the expression frequency of CD4^+^Foxp3^+^ in severe EV71 infection but also reduced the plasma levels of cytokines, IL-6, IL-8 and IL-10. cAMP is synthesized from ATP by adenylyl cyclase and degraded by PDE. Possible explanations for low plasma cAMP levels in severe EV71 infections include decreased substrates for tissue ATP generation, mitochondrial dysfunction or damage, decreased or inhibited adenyl cyclase activity, and increased phosphodiesterase activity. Furthermore, protein kinase A (PKA) and exchange proteins directly activated by cAMP (Epac) mediate most of the cAMP-regulated physiological functions [38]. The functions and mechanisms on decreasing Treg and cAMP in severe EV71 infection deserved further study.

Elevated cAMP levels are associated with the suppression of innate immune functions, including the production of proinflammatory mediators. cAMP is capable of suppressing production of TNF-α and IL-12 [37]. The transcription factor c-Fos was responsible for the cAMP-mediated suppression of inflammatory cytokine production [36]. Additionally, cAMP inhibits T cell activation and proliferation, and is likely mediated by Tregs [39]. Klein et al. showed that both the suppressive activity and functional state of Treg strictly correlate with intracellular cAMP levels, and can be manipulated by interfering with the function of cAMP-producing (ie, adenyl cyclase) and cAMP-degrading (ie, PDE) enzymes [40]. These findings may provide partial explanation to the increased Treg expression and plasma level of cAMP, and decreased plasma levels of cytokines in severe EV71–infected patients with milrinone treatment.

Furthermore, Yang et al. reported that patients with EV71 meningoencephalitis exhibited a higher frequency of polymorphism of CTLA-4 at position 49 of exon 1 in the G/G genotype [41]. They suggested that CTLA-4 polymorphism might be associated with EV71 meningoencephalitis in younger children. CTLA-4 is constitutively expressed on Tregs. cAMP-elevating agents induced upregulation of soluble and membrane isoforms of the inhibitory molecule CTLA-4 in resting T lymphocytes [42]. These evidences also supported the notions that milrinone treatment enhanced level of cAMP may play a key role in modulating the immune response to severe EV71 infection by changing Treg and cytokines expression.

It is more plausible that in human EV71 infection, Treg cells are one of the essential factors in controlling the inflammatory process. PDE inhibitors, milrinone, that prevent rapid degradation of cAMP should be a valuable agent for the improvement of Treg-mediated suppression. An improved understanding of its regulatory mechanisms of the changes of cAMP levels and Treg expression may be a promising way to shape adaptive immune responses and new therapeutic strategies to EV71 infection.
